# Non-diabetic Ketoacidosis in a Patient With Amyotrophic Lateral Sclerosis: The Role of Stewart’s Approach in Analyzing Acid-Base Disorders

**DOI:** 10.7759/cureus.82585

**Published:** 2025-04-19

**Authors:** Frédéric Franconieri, Sophie Fayolle, Prescillia Raviol

**Affiliations:** 1 Internal Medicine, Centre Hospitalier du Forez, Montbrison, FRA

**Keywords:** acid-base disorders, amyotrophic lateral sclerosis, keto acidosis, protein energy malnutrition (pem), stewart approach

## Abstract

Amyotrophic lateral sclerosis (ALS) is often complicated by severe malnutrition, increasing the risk of metabolic disturbances. Non-diabetic ketoacidosis (NDKA) is a rare but serious complication, typically related to prolonged fasting or catabolic states. A 62-year-old female patient with ALS and hypothyroidism presented with pneumonia and tetraplegia. Her body mass index (BMI) was 17 kg/m². Laboratory findings showed a high anion gap (AG) metabolic acidosis (pH 7.23, bicarbonate 13 mmol/L, partial pressure of carbon dioxide (pCO₂) 28 mmHg) without hyperlactatemia, but with significant ketonemia (5 mmol/L), severe hypophosphatemia, and signs of systemic inflammation. Upon admission, she received an intravenous infusion of 4.2% sodium bicarbonate. The simplified strong ion difference (SID) was preserved, excluding dilutional or hyperchloremic causes. Stewart’s physicochemical approach, supported by a Gamblegram, revealed an acidosis due to unmeasured fixed acids, specifically ketone bodies. In light of this, bicarbonate therapy was discontinued, and nutritional correction with glucose hydration led to rapid clinical and biochemical improvement. This case illustrates the diagnostic and therapeutic value of Stewart’s model in complex acid-base disturbances and underscores the need for early nutritional assessment in ALS patients. To our knowledge, this is the first reported case of NDKA in ALS, highlighting a rare but clinically relevant metabolic complication.

## Introduction

Amyotrophic lateral sclerosis (ALS) is a severe neurodegenerative disease, often associated with metabolic complications, including severe malnutrition [1]. These nutritional imbalances can lead to complex acid-base disturbances [2]. Acid-base disorders are traditionally interpreted using the Henderson-Hasselbalch equation, which relates blood pH to the ratio of bicarbonate concentration to the partial pressure of carbon dioxide (pCO₂) [3]. Although widely used for its clinical practicality, this model simplifies the complexity of acid-base physiology by focusing on two dependent variables without accounting for the physicochemical mechanisms that govern their behavior. In contrast, Stewart’s approach offers a mechanistic framework based on four core principles: electroneutrality, water dissociation into H⁺ and OH⁻, the conservation of mass, and the dissociation equilibrium of weak acids (AH ⇌ A⁻ + H⁺). According to this model, pH is determined by three independent variables: the strong ion difference (SID), the pCO₂, and the concentration of weak acids. This approach provides a more physiologically coherent understanding of acid-base balance, particularly in complex clinical situations where multiple processes coexist [4,5].

We report here a rare case of non-diabetic ketoacidosis (NDKA) in a patient with ALS, highlighting the diagnostic value of Stewart’s method in elucidating the underlying pathophysiological mechanism and guiding appropriate therapeutic decisions.

## Case presentation

A 62-year-old female patient, diagnosed with ALS in 2021, presented with tetraplegia and hypothyroidism, for which she was being treated with L-thyroxine and riluzole. She was admitted to the emergency department for infectious pneumonia. 

Upon examination, she exhibited tachypnea (28 breaths per minute), a temperature of 37.9°C, and an oxygen saturation (SpO₂) of 91% on room air. Her body mass index (BMI) was 17 kg/m². Laboratory tests revealed a major inflammatory syndrome, with a markedly elevated C-reactive protein (CRP; 387 mg/L), leukocytosis (18.8 G/L, 90% neutrophils), and anemia (hemoglobin 11 g/dL). She also had profound hypophosphatemia (0.43 mmol/L), hypomagnesemia (0.70 mmol/L), and low prealbumin (0.1 g/L), consistent with severe malnutrition and systemic inflammation. A metabolic acidosis with a low bicarbonate level (13 mmol/L), decreased pH (7.23), and compensatory hypocapnia (pCO₂ 28 mmHg) was observed, without hyperlactatemia (lactate 0.7 mmol/L). Additional values included platelet count 350 G/L, sodium 137 mmol/L, potassium 3.6 mmol/L, chloride 102 mmol/L, creatinine 40 μmol/L, urea 3 mmol/L, fasting glucose 0.8 g/L, glycated hemoglobin (HbA1c) 5.2%, and albumin 38 g/L. The patient's laboratory results are summarized in Table 1.

**Table 1:** The patient's laboratory results on admission to the emergency department

Parameter	Patient value	Normal range
pH	7.23	7.35 – 7.45
Partial pressure of carbon dioxide (PCO_2_), mmHg	28	35 -45
Partial pressure of oxygen (PO_2_) mmHg	70	75 - 100
Bicarbonate (HCO3), mmol/L	13	22 - 24
Sodium, mmol/L	137	135 - 145
Chloride, mmol/L	102	95 - 105
Potassium, mmol/L	3.6	3.5 -5
Calcium, mmol/L	2.55	2.2 – 2.6
Phosphorus, mmol/L	0.43	0.8 – 1.45
Magnesium, mmol/L	0.70	0,74 – 1,07
Glucose, g/L	0.8	0.7 – 1.1
Albumin, g/L	38	35 - 45
Prealbumin, g/L	0.1	0,2 – 0,4
Creatinine, μmol/L	40	50 - 100
Urea, mmol/L	3	2,5 – 7
Lactate, mmol/L	0.7	0.5 – 1.5
C-reactive protein, mg/L	387	0 – 5
Hemoglobin, g/dL	11	M: 13 – 17 F: 12 - 15
White cell count, G/L	18.8	4 - 10
Platelet count, G/L	350	150 - 400

Upon her arrival at our department, the patient had already received an intravenous infusion of 4.2% bicarbonate. To further characterize the acid-base disturbance, both the traditional anion gap (AG) and the simplified apparent SID (SIDsa) were evaluated. The AG, calculated as AG = [Na⁺] - [Cl⁻] - [HCO₃⁻], was elevated at 22 mmol/L (normal range: 8-16 mmol/L), indicating the presence of unmeasured anions. In parallel, the SIDsa was calculated as [Na⁺] + [K⁺] - [Cl⁻] - [lactate], yielding a value of 42 mEq/L, within the normal range. This excluded a reduction in SID and supported the diagnosis of a high AG metabolic acidosis due to the accumulation of unmeasured anions. 

**Figure 1:**
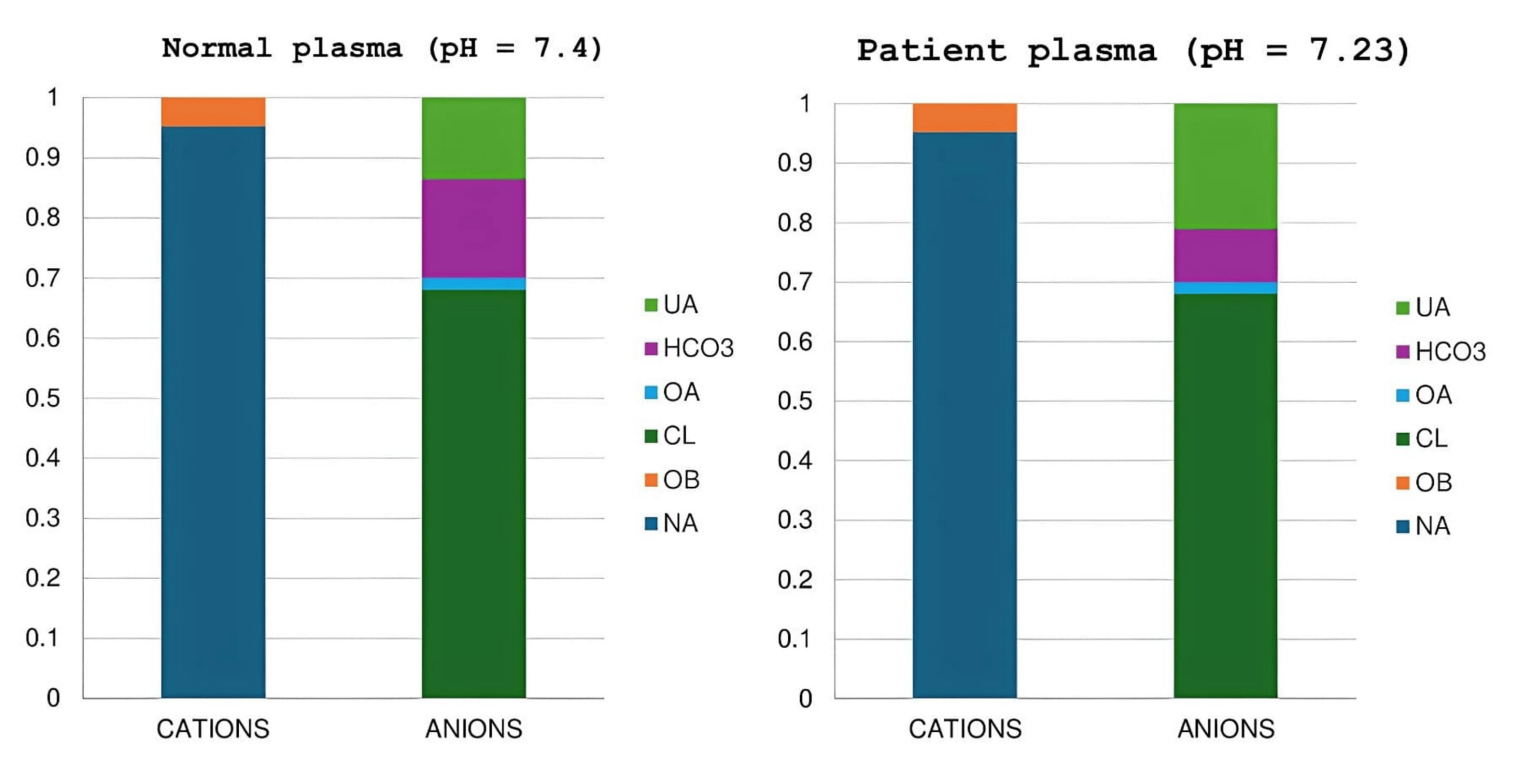
A Gamblegram of normal plasma and the patient's plasma UA: unmeasured anions; HCO3: bicarbonate; OA: other measurable anions (lactates, phosphorus, albumin); CL: chloride; OB: other bases (magnesium, calcium, potassium); NA: sodium

To facilitate a comprehensive interpretation of the acid-base profile, a Gamblegram was constructed, juxtaposing the patient's plasma ionic composition with a normative reference. This graphical representation underscored a marked reduction in bicarbonate, offset by a proportional increase in unmeasured anions, while the concentrations of measured strong ions remained within physiological ranges. The preserved SID ruled out a dilutional or hyperchloremic mechanism, reinforcing the diagnosis of a high AG metabolic acidosis due to unmeasured fixed acids (Figure 1).

Measurement of ketone bodies revealed a ketonemia of 5 mmol/L. A diagnosis of NDKA, secondary to malnutrition and prolonged fasting, was established. Bicarbonate therapy was discontinued, and treatment with glucose hydration and gradual refeeding led to rapid correction of acidosis.

## Discussion

This case emphasizes the importance of comprehensive acid-base analysis in complex clinical settings. As previously outlined, Stewart’s approach defines acid-base balance through three independent variables: SID, pCO₂, and the concentration of weak acids, each governed by core physicochemical principles [4,5]. This model departs from the traditional Henderson-Hasselbalch equation by treating bicarbonate not as a primary regulator, but as a dependent variable influenced by these three determinants [6,7].

Stewart’s method proves especially helpful in clinical contexts marked by overlapping physiological disturbances [8]. In our patient, it enabled a more accurate interpretation of a seemingly straightforward acidosis by revealing the presence of unmeasured anions. The normal SID and absence of hyperchloremia or lactate pointed toward a high AG metabolic acidosis, ultimately attributed to ketone body accumulation in a context of severe malnutrition.

The Gamblegram further illustrated this mechanism, showing a decrease in bicarbonate concentration offset by the presence of unmeasured fixed acids. In this starvation-induced ketoacidosis, hepatic overproduction of ketone bodies, primarily beta (β)-hydroxybutyrate and acetoacetate, resulted in the accumulation of strong anions in plasma [9]. This accumulation led to increased hydrogen ion concentration through water dissociation, which consumed bicarbonate and ultimately lowered pH. Differential diagnoses, including renal failure, lactic acidosis, and toxic ingestions, were ruled out based on clinical and laboratory findings [10,11].

The apparent SID is traditionally calculated as [Na⁺] + [K⁺] + [Ca²⁺] + [Mg²⁺] - [Cl⁻] - [lactate], with normal values around 40 mEq/L [12]. At the bedside, a simplified version, [Na⁺] + [K⁺] - [Cl⁻] - [lactate], is commonly used, with a normal reference of approximately 42 mEq/L. The chloride-to-sodium ratio (Cl⁻/Na⁺), normally 76% ± 3%, can help detect subtle chloride disturbances, including those masked by dilutional or concentration effects in cases of dysnatremia [13]. A ratio >79% suggests relative chloride excess, while <73% points toward chloride loss. In our patient, the ratio was 74.5%, indicating no hyperchloremic contribution to the acidosis. Together, these values supported the hypothesis of an AG metabolic acidosis [14,15]. The normal SID (42 mEq/L) and normal lactate level quickly suggested an excess of unmeasured anions. This was confirmed by elevated ketone bodies, consistent with NDKA. The Gamblegram clearly showed a bicarbonate drop, replaced by unmeasured anions, while other electrolytes remained stable, confirming fixed acid-driven acidosis.

Hypophosphatemia, likely related to severe malnutrition and possibly early refeeding [16], may have also contributed to metabolic alkalosis by reducing the amount of weak acids. Moreover, hypophosphatemia may affect respiratory muscle performance by impairing adenosine triphosphate (ATP) synthesis, potentially aggravating the respiratory status [17]. 

This analytical framework is particularly valuable in critical care settings, where patients often exhibit overlapping acid-base imbalances. Metabolic acidosis (from lactate buildup or renal failure, for example), metabolic alkalosis (due to hypoalbuminemia or hypophosphatemia, for example), and concurrent respiratory disturbances frequently coexist, sometimes offsetting each other. These complex acid-base disorders can result in a deceptively normal pH, leading to misinterpretation if only traditional analysis is applied. Stewart’s model offers a more coherent physiological understanding in such multifactorial contexts [18]. Our patient exemplifies this complexity. Her ALS-associated malnutrition, prolonged fasting, dysphagia, hypomobility due to tetraplegia, and muscle wasting created a catabolic state conducive to starvation ketoacidosis. The superimposed pulmonary infection likely acted as a precipitating factor, increasing metabolic demand, worsening catabolism, and further reducing oral intake. 

In clinical practice, the administration of intravenous bicarbonate is a common first-line response in the face of metabolic acidosis. However, this approach is not always appropriate and can even be counterproductive if the underlying cause is not adequately identified. In our patient, the acidosis was not due to a deficit of bicarbonate per se, but rather to an accumulation of unmeasured anions related to malnutrition and fasting. Administering bicarbonate in this context risked further reducing the SID, potentially aggravating the acidosis and obscuring the need for causal treatment. By using Stewart’s method, which integrates all physicochemical contributors to acid-base balance, we were able to propose a more targeted therapeutic strategy. Rather than buffering the symptom, we addressed the cause: nutritional correction and glucose infusion led to a rapid normalization of pH and clinical improvement. This case illustrates how a mechanistic, Stewart-based analysis allows for more physiologically coherent and causally appropriate treatment decisions in acid-base disorders.

A thorough literature review on PubMed using Medical Subject Headings (MeSH) terms such as “Amyotrophic lateral sclerosis and ketoacidosis,” “Amyotrophic lateral sclerosis and metabolic acidosis,” “Starvation-induced ketoacidosis and ALS,” and “(Amyotrophic Lateral Sclerosis OR ALS) AND (Ketoacidosis OR Non-diabetic ketoacidosis OR Starvation ketoacidosis)”) revealed no similar cases. To the best of our knowledge, this is the first description of NDKA in a patient with ALS.

## Conclusions

This case highlights a rare but serious metabolic complication of ALS: NDKA induced by malnutrition. Stewart’s approach, though underutilized in routine practice, proved instrumental in diagnosing and understanding the pathophysiology of the acidosis. By shifting the focus from bicarbonate levels to the underlying ionic mechanisms, Stewart's approach not only prevented unnecessary bicarbonate therapy but also guided a more targeted and individualized treatment plan. This approach should be more widely integrated into the evaluation of acid-base disturbances, particularly in malnourished or neurologically impaired patients. Early nutritional assessment and individualized management are essential to prevent such complications and improve outcomes in ALS and similarly vulnerable populations.
